# The Loss of Cellular Junctions in Epithelial Lung Cells Induced by Cigarette Smoke Is Attenuated by Corilagin

**DOI:** 10.1155/2015/631758

**Published:** 2015-02-24

**Authors:** Ximena M. Muresan, Franco Cervellati, Claudia Sticozzi, Giuseppe Belmonte, Chung Hin Chui, Ilaria Lampronti, Monica Borgatti, Roberto Gambari, Giuseppe Valacchi

**Affiliations:** ^1^Department of Life Sciences and Biotechnology, University of Ferrara, 44121 Ferrara, Italy; ^2^Clinical Division, School of Chinese Medicine, Hong Kong Baptist University, Kowloon Tong, Hong Kong; ^3^Biotechnology Center, University of Ferrara, 44121 Ferrara, Italy

## Abstract

Cigarette smoke (CS) contains over 4700 compounds, many of which can affect cellular redox balance through free radicals production or through the modulation of antioxidant enzymes. The respiratory tract is one of the organs directly exposed to CS and it is known that CS can damage the integrity of lung epithelium by affecting cell junctions and increasing epithelium permeability. In this study, we have used a human lung epithelial cell line, Calu-3, to evaluate the effect of CS on lung epithelial cell junctions levels, with special focus on the expression of two proteins involved in intercellular communication: connexins (Cx) 40 and 43. CS exposure increased Cx40 gene expression but not of Cx43. CS also induced NF*κ*B activation and the formation of 4HNE-Cxs adducts. Since corilagin, a natural polyphenol, is able to inhibit NF*κ*B activation, we have determined whether corilagin could counteract the effect of CS on Cxs expression. Corilagin was able to diminish CS induced Cx40 gene expression, 4HNE-Cx40 adducts formation, and NF*κ*B activation. The results of this study demonstrated that CS induced the loss of cellular junctions in lung epithelium, possibly as a consequence of Cx-4HNE adducts formation, and corilagin seems to be able to abolish these CS induced alterations.

## 1. Introduction

Cigarette smoke (CS) is a heterogeneous mixture formed by a gaseous phase and solid particles, and it contains more than 4700 compounds, including carcinogen and oxidant substances [1, 2]. Due to its ability to induce cellular oxidative stress, CS causes noxious effects in a large part of human organs, such as cardiovascular system [3], cutaneous tissues [4], retina [5], and of course lung tissues. The ability of CS to induce oxidative stress has been associated with cellular damage such as DNA mutations [6], lipid peroxidation with formation of reactive aldehydes [7], and proteins oxidation [8]. In addition, it is documented that CS is able to promote inflammatory responses in cells, via TNF-*α* and NF*κ*B signaling pathway [9].

The main target of CS is obviously the respiratory tract characterized by a pseudostratified epithelium, which acts as a barrier protecting the organism from environmental antigens. Respiratory epithelial cells are bound together by different cellular junctions, as described in Heijink et al. [10]. Gap junctions, in particular, are membrane channels formed by the union of two hemichannels belonging to adjacent cells; these hemichannels are also known as connexons; each of them is composed of six connexins that are four-pass transmembrane proteins [11]. Gap junctions have multiple functions, by allowing the direct passage of ions, second messengers, and metabolites among cells. Therefore, gap junctions allow an essential communication that maintains normal functions of tissues and organs [12]. These communications are important in all physiological cellular processes, such as cell growth, cell development, cell differentiation, and cell death, and its absence leads to changes in the cellular homeostasis [13]. In addition, connexins are known to affect cell migration and adhesion [14–16].

In the last few decades, many studies have shown that polyphenols and oxidant scavengers can contrast CS induced oxidative stress [17–20]. Corilagin is a polyphenol, member of the tannin family extracted from different plants like* Dimocarpus longan* [21] and* Geranium thunbergii* [22]. This natural compound seems to have beneficial effects in several cardiovascular disorders, hypertension, thrombosis, or atherosclerosis [23], but it is also known for its antiproliferative and antitumor effects [24]. Zhao et al. have demonstrated the anti-inflammatory properties of corilagin. Their study shows that corilagin is able to block NF*κ*B activation and its nuclear translocation, demonstrating anti-inflammatory characteristic. In addition, it has been shown that corilagin decreases the production of proinflammatory cytokines like TNF-*α*, IL-1*β*, IL-6, iNOS, and COX-2 [25]. Furthermore, some studies have evidenced the ability of corilagin to inhibit IL-8 production by blocking TNF-*α* induced NF*κ*B nuclear translocation [26]. It has been also demonstrated that corilagin is hepatoprotective as it acts like radical scavenger for superoxide anion and peroxyl radicals. In addition corilagin inhibits ROS production from leukocytes as well as free radicals formation and lipid peroxidation in mitochondria [27].

In our study we evaluated the damaging effects of CS on lung epithelial cells; in particular we investigated CS-modulation of gap junctions and connexin expression and we also determined whether corilagin could counteract the noxious effect of CS. For our purpose, we have used Calu-3 cell line grown in air-liquid interface, which has been reported to be a good model to study biological agents on human airway epithelial cells functions and structures. Indeed this model has been extensively used for lung pathophysiology and therapeutics studies and it exhibits many characteristics of the primary airway cell cultures thanks to its ability to form stable cell-to-cell junctions [28].

## 2. Materials and Methods

### 2.1. Cell Culture and Treatment

Calu-3 cells cultured in 75 cm^2^ plastic flasks (BD Falcon, USA) were grown in Dulbecco's modified Eagle's medium (Lonza, Milan, Italy) supplemented with 10% fetal bovine serum (EuroClone, Milan, Italy), 2 mM l-glutamine (Lonza, Milan, Italy), and antibiotics (100 UI/mL penicillin and 100 *μ*g/mL streptomycin) (Lonza, Milan, Italy). They were maintained at 37°C in a humidified 5% CO_2_ atmosphere. Upon reaching 90% confluence, cells were harvested using 0,02% trypsin/EDTA (Lonza, Milan, Italy). After trypsinisation, Calu-3 cells were seeded at a density of 1 × 10^6^ cells/mL onto 0.4 *μ*m pore size, 4.15 cm^2^ surface area polyethylene terephthalate Transwell cell culture inserts (BD Falcon, USA), placed in 6-well cell culture plates (BD Falcon, USA). When the cells reached 90% of confluence and were pretreated with corilagin 10 *μ*M for 24 hr, then the culture media were changed and the cells were exposed to CS.

Corilagin was a gift by the China National Institute for the Control of Pharmaceutical and Biological Products. The powder was dissolved in ethanol and the 10 *μ*M solution was added to the cells (final ethanol concentration 0.1%).

### 2.2. CS Exposure

Cells were exposed to CS for 50 min in fresh serum-free media (as previously described [29]) while control cells were exposed to filtered air (50 min). CS was generated by burning one research cigarette (12 mg tar, 1.1 mg nicotine) using a vacuum pump to draw air through the burning cigarette and leading the smoke stream over the cell culture as described previously [29]. After the exposure (air or CS), fresh media supplemented with 10% FBS were added to the cells.

### 2.3. Cytotoxicity Determination

After CS exposure, culture media were collected at different time points (50 min, 6 hr, 12 hr, and 24 hr). Cytotoxicity was determined by LDH release in the media, measured by enzymatic assay; in the first step NAD^+^ is reduced to NADH/H^+^ by the LDH-catalyzed conversion of lactate to pyruvate; in the second step the catalyst (diaphorase) transfers H/H^+^ from NADH/H^+^ to tetrazolium salt which is reduced to formazan. The amount of LDH in the supernatant was determined and calculated according to kit instructions (EuroClone Milan, Italy). All tests were performed in triplicate and assays were repeated three times independently.

### 2.4. Protein Extraction

At each time point cells were washed with ice-cold PBS and lysed in ice-cold lysis buffer (20 mM Tris pH 8, 150 mM NaCl, 1% Triton X-100, 1 mM sodium orthovanadate, 1 *μ*g/mL leupeptin, 1 *μ*g/mL aprotinin, 1 *μ*g/mL pepstatin, 10 *μ*g/mL PMSF, and 5 mM *β*-glycerophosphate) (Sigma, Milan, Italy). After centrifugation (13,500 rpm, 15 min at 4°C), the supernatants were collected. Proteins concentration was determined by the method of Bradford (BioRad, Milan, Italy).

### 2.5. Western Blot Analysis

Sixty *μ*g boiled protein was loaded onto 10% sodium dodecyl sulphate-polyacrylamide electrophoresis gels and separated by molecular size. Gels were electroblotted onto nitrocellulose membranes and then were blocked for 1 hr in Tris-buffered saline, pH 7.5, containing 0.5% Tween 20 and 3% milk. Membranes were incubated overnight at 4°C with the appropriate primary antibodies: Cx40 (Santa Cruz Biotechnology Inc., USA), Cx43 (Santa Cruz Biotechnology Inc., USA), 4HNE (Chemicon, USA), and *β*-actin (Cell Signalling, Celbio, Milan, Italy). The membranes were then incubated with horseradish peroxidase-conjugated secondary antibody for 1 hr, and the bound antibodies were detected by chemiluminescence (BioRad, Milan, Italy). *β*-actin was used as loading control. Images of the bands were digitized and the densitometry analysis was performed using ImageJ software.

### 2.6. Electrophoretic Mobility Shift Assay (EMSA)

Electrophoretic Mobility Shift Assay (EMSA) was performed as previously described [30, 31]. Briefly, double-stranded synthetic oligodeoxynucleotides mimicking two functional NF*κ*B binding sites displaying different sequence (A, sense: 5′-GTT CTG GGA TTT CCC CCG AT-3′; B, sense: 5′-CAG CAG GAA CGT CCC AGA GAA-3′) [32] have been employed. Oligodeoxynucleotides were labeled with *γ* ^32^-P-ATP using 10 units of T4-polynucleotide-kinase (KinaseMAX, Ambion) in 500 mM Tris-HCl, pH 7.6, 100 mM MgCl_2_, 50 mM DTT, 1 mM spermidine, and 1 mM EDTA in the presence of 50 mCi *γ* ^32^-P-ATP in a volume of 20 *μ*L for 60 minutes at 37°C. Reaction was brought to 150 mM NaCl and 150 ng complementary oligodeoxynucleotide was added. Reaction temperature was increased to 100°C for 5 minutes and left diminishing to room temperature overnight. Binding reactions were set up as described elsewhere [30] in a total volume of 20 *μ*L containing buffer TF plus 5% glycerol, 1 mM dithiothreitol, 5 ng of human NF*κ*B p50 protein, and different concentrations of corilagin. After incubation of 20 min at room temperature, 0.25 ng of ^32^P-labeled oligonucleotides was added to the samples for further 20 min at room temperature and then they were electrophoresed at constant voltage (200 V) under low ionic strength conditions (0.25x TBE buffer: 22 mM Tris borate, 0.4 mM EDTA) on 6% polyacrylamide gels. Gels were dried and subjected to standard autoradiographic procedures [30].

### 2.7. Protein Carbonyls

The levels of proteins carbonyl groups were determined by OxyBlot (Chemicon, USA). Briefly, after derivatization of carbonyl groups to dinitrophenylhydrazone (DNP-hydrazone) by reaction with dinitrophenylhydrazine (DNPH), the DNP-derivatized protein samples were separated by polyacrylamide gel electrophoresis followed by Western blotting as previously described [33].

### 2.8. Immunoprecipitation

Cell lysates containing 300 *μ*g of protein were mixed with Dynabeads protein G (Invitrogen, USA) and 2 *μ*g of polyclonal antibody against Cx40. Following immunoprecipitation of Cx40, the proteins were separated by SDS-PAGE, electrotransferred in a nitrocellulose membranes, and immunoblotted with 4HNE antibody.

### 2.9. Preparation of Cytoplasmic and Nuclear Extracts for Western Blotting

For cytoplasmic and nuclear extracts, cells were resuspended in 100 *μ*L of hypotonic buffer containing 10 mmol/L HEPES (pH 7.9), 10 mmol/L KCl, 1.5 mmol/L MgCl_2_, 0.3% Nonidet P-40, 0.5 mmol/L dithiothreitol, 0.5 mmol/L phenylmethylsulphonylfluoride, protease inhibitor cocktail, 1 mmol/L orthovanadate, and 5 mmol/L *β*-glycerophosphate. The lysates were incubated for 15 min on ice and then centrifuged at 1500 ×g for 5 min at 4°C for collection of the supernatant containing cytosolic proteins. Pellets containing nuclei were resuspended in 50 *μ*L of extraction buffer containing 20 mmol/L HEPES (pH 7.9), 1.5 mmol/L MgCl_2_, 0.6 mol/L KCl, 0.2 mmol/L EDTA, 20% glycerol, 0.5 mmol/L phenylmethylsulfonyl fluoride, protease inhibitor cocktail, 1 mmol/L orthovanadate, and 5 mmol/L *β*-glycerophosphate and then were incubated for 30 min on ice. Samples were then centrifuged at 13000 rpm for 15 min to obtain supernatants containing nuclear fractions. Protein samples were separated by polyacrylamide gel electrophoresis followed by Western blotting. NF*κ*B (Santa Cruz Biotechnology Inc., USA) was used as primary antibody.

### 2.10. RT-qPCR (Reverse Transcription Quantitative Real-Time PCR)

Total RNA from each sample was extracted with the AURUM Total RNA Mini Kit with DNAse digestion (Bio-Rad Laboratories Inc., USA), according to the manufacturer's recommended procedure. After solubilization in RNAase-free water, total RNA was quantified by Bio-Rad SmartSpec Plus spectrophotometer (Bio-Rad Laboratories Inc., USA). First-strand cDNA was generated from 1 *μ*g of total RNA using iScript cDNA Synthesis Kit (Bio-Rad Laboratories Inc., USA). Primer pairs were obtained from PrimerBank from the Real-Time PCR Primer and Probe Database, RTPrimerDB, to hybridise to unique regions of the appropriate gene sequence. The reverse transcriptase (RT) PCR reactions were carried out using 1 *μ*L of cDNA in a 15 *μ*L total volume of PCR buffer (Invitrogen, Milan, Italy), containing 3 mM MgCl_2_, 300 *μ*M dNTPs, and 300 nM of appropriate primers. Taq polymerase (0.35 U) was also added. The amplification reactions were carried out in a thermal gradient cycler (Bio-Rad Laboratories Inc., USA) for 40 cycles. Each cycle consists of denaturation for 30 s at 94°C, annealing for 30 s at 60°C, and extension for 30 s at 72°C. A final extension step at 72°C for 5 min terminates the amplification. For each amplification, two controls were performed: (i) RT-PCR mixture with no reverse transcriptase to control for genomic DNA contamination and (ii) PCR mixture with no cDNA template, to check for possible external contamination. A 5 *μ*L sample of the PCR reaction was electrophoresed on an ethidium bromide containing 2% agarose gel by the use of the Bio-Rad Subcell GT system. Quantitative Real-Time PCR (qPCR) was performed using SYBR Green on iQ5 Multicolor Real-Time PCR Detection System (Bio-Rad Laboratories Inc., USA). The final reaction mixture contained 1 *μ*L of cDNA, 300 nM of each primer, 7.5 *μ*L of iQ SYBR Green Supermix (Bio-Rad Laboratories Inc., USA), and RNAse-free water to complete the reaction mixture volume to 15 *μ*L. All reactions were run as triplicates. The QPCR was performed with a hot-start denaturation step at 95°C for 3 min and then was carried out for 40 cycles at 95°C for 10 s and at 60°C for 20 s. The fluorescence was read during the reaction by the Opticon Monitor 3 software (Bio-Rad Laboratories Inc., USA), allowing a continuous monitoring of the amount of PCR products. Primers are initially used to generate a standard curve over a large dynamic range of starting cDNA quantity which allows calculating the amplification efficiency (a critical value for the correct quantification of expression data) for each of the primer pairs. The melting curve analysis was performed at the end of each experiment to verify that a single product for primer pair is amplified (data not shown). As to control experiments, gel electrophoresis was also performed to verify the sizes of the amplified QPCR products. Ribosomal proteins L13a (RPL13a), L11a (RPL11a), and GAPDH were used in our experiments as internal standards. Samples were compared using the relative cycle threshold (CT) method. The fold increase or decrease was determined relative to a control after normalising to RPL13a (internal standard). The formula 2 − ΔΔCT was used, where ΔCT is gene of interest CT (RPL13A CT) and ΔΔCT is ΔCT experimental (ΔCT control).

### 2.11. Ultrastructural Analysis

Cells were scraped and collected in 0.1 M cacodylate buffer (pH 7.4) and then spun in 1.5 mL tubes at 2000 ×g for 5 min. Pellets were fixed with 2.5% glutaraldehyde in 0.1 M sodium cacodylate buffer for 4 hr at 4°C. They were then washed with 0.1 M cacodylate buffer (pH 7.4) three times and postfixed in 1% osmium tetroxide and 0.1 M cacodylate buffer at pH 7.4 for 1 hr at room temperature. The specimens were dehydrated in graded concentrations of ethanol and embedded in epoxide resin (Agar Scientific, 66A Cambridge Road, Stansted Essex CM24 8DA, UK).

Cells were then transferred to latex modules filled with resin and subsequently thermally cured at 60°C for 48 hr.

Semithin sections (0.5–1 *μ*m thickness) were cut using an ultramicrotome (Reichard Ultracut S, Austria) stained with toluidine blue, and blocks were selected for thinning. Ultrathin sections of about 40–60 nm were cut and mounted onto formvar-coated copper grids. These were then double-stained with 1% uranyl acetate and 0.1% lead citrate for 30 min each and examined under a transmission electron microscope, Hitachi H-800 (Tokyo, Japan), at an accelerating voltage of 100 KV.

### 2.12. Statistical Analysis

For each of the variables tested, two-way analysis of variance (ANOVA) was used. A significant result was indicated by a *P* value < 0.05. Data are expressed as mean ± SD of triplicate determinations obtained in 5 independent experiments.

## 3. Results

### 3.1. Cytotoxic Effect of CS on Human Lung Epithelial Calu-3 Cells

**Figure 1:**
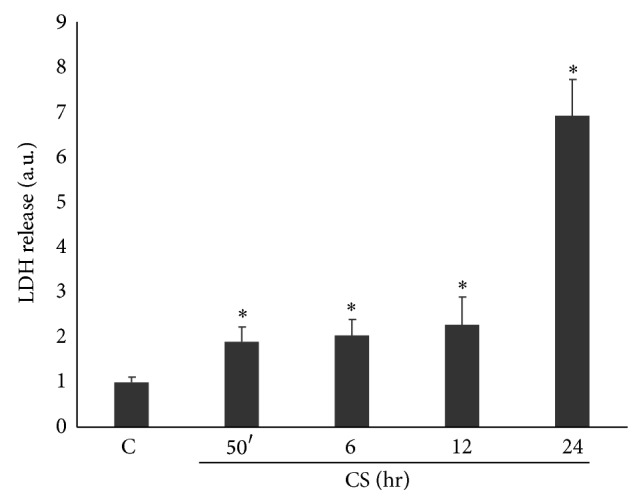
CS induces toxicity in Calu-3 cells. Cells were exposed to CS for 50 minutes and media were collected at different time points (50 min–24 hr). The graph shows evidence of toxicity in cells exposed to CS as measured by LDH release in the media. Data shown are representative of five independent experiments. *P* < 0.05; ∗ versus C.

The first step of the study was to evaluate Calu-3 cytotoxicity induced by CS. After CS exposure, media were collected at different time points (from 50 min to 24 hr) and the lactate dehydrogenase (LDH) release from the cells was measured. As shown in Figure 1, CS induced a significant release of LDH already after 50 min of CS exposure (2-fold) and this effect was even more pronounced after 24 hr leading to 7-fold increase of LDH release with respect to the air exposed cells (the same results were obtained by trypan blue exclusion assay, Table 1).

### 3.2. CS Exposure Induced Cells Detachment

**Figure 2:**
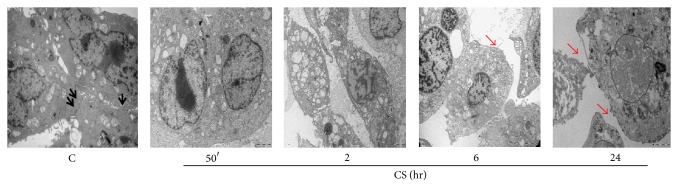
Cells lose intercellular contact after CS exposure. Control cells are well assembled and show the presence of densifications in the contact regions between them (see black arrows). At later time points (12 and 24 hr) the loss of cell-to-cell contact is clear as shown by red arrows. Pictures are taken at different magnifications.

Ultrastructural study was performed to better evaluate the status of the cells exposed to CS and by transmission electronic microscopy (TEM). As shown in Figure 2, control cells (C) appeared with a normal cellular structure and a clear cell-to-cell contact. After CS exposure the cells started to form internal vesicles (50 min, 2 hr) and to lose intercellular contacts in a time-dependent manner (6 and 24 hr), as evidenced by the red arrows in the representative image.

### 3.3. Evidence of Oxidized Proteins in Calu-3 Cells Exposed to CS

**Figure 3:**
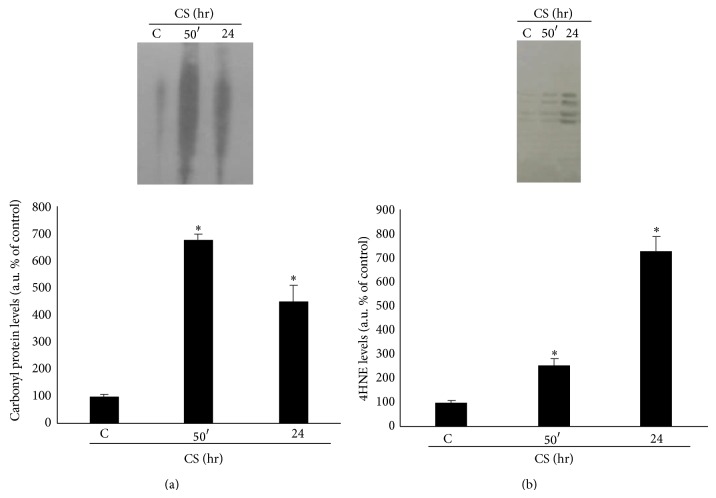
CS promotes proteins oxidation and lipid peroxidation. WBs in the top panel are representative of five independent experiments. Quantification of carbonyl proteins is shown in the bottom left panel (a) and quantification of 4HNE is shown in the bottom right panel (b). Data are expressed as arbitrary units (average of five independent experiments; *P* < 0.05; ∗ versus C).

Calu-3 cells exposed to CS showed increase levels of oxidative stress as determined by the protein oxidation and lipid peroxidation products formation. Carbonyl proteins (Figure 3(a)) levels were significantly higher immediately after CS exposure, reaching the highest levels 50 minutes after CS exposure. In addition, CS induced the formation of 4-hydroxynonenal (4HNE) protein adducts (Figure 3(b)) after CS exposure in a time-dependent manner (2-fold at 50 min and 6-fold at 24 hr).

### 3.4. CS Induced the Activation of NF*κ*B

**Figure 4:**
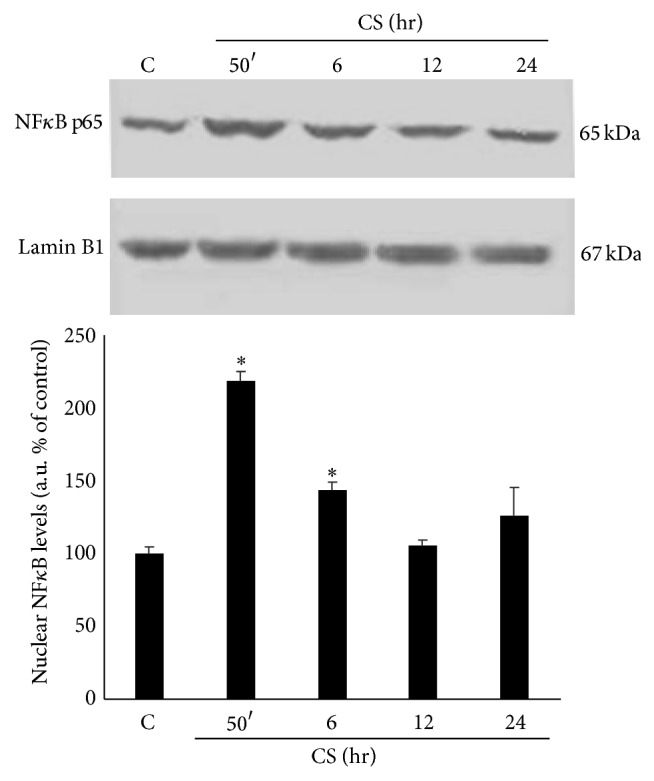
CS exposure activates NF*κ*B. WB in the top panel is representative of five independent experiments. In the bottom panel is shown the quantification of nuclear NF*κ*B protein levels. Data are expressed as arbitrary units (average of five independent experiments; *P* < 0.05; ∗ versus C). Lamin B1 was used as loading control.

As NF*κ*B activation is determined by the translocation of p65/p50 subunits to the nucleus, Western blotting for p65 subunit was performed on nuclear protein extracts from Calu-3 cells exposed to CS. As shown in Figure 4, CS determined a 2-fold increase in p65 nuclear protein levels at the earlier time points after CS exposure. On the contrary, no evidence of NF*κ*B activation was found at the later time points (i.e., 12 and 24 hr exposure), suggesting an early NF*κ*B activation by CS exposure.

### 3.5. Corilagin Reduces the Cytotoxicity Induced by CS Exposure

**Figure 5:**
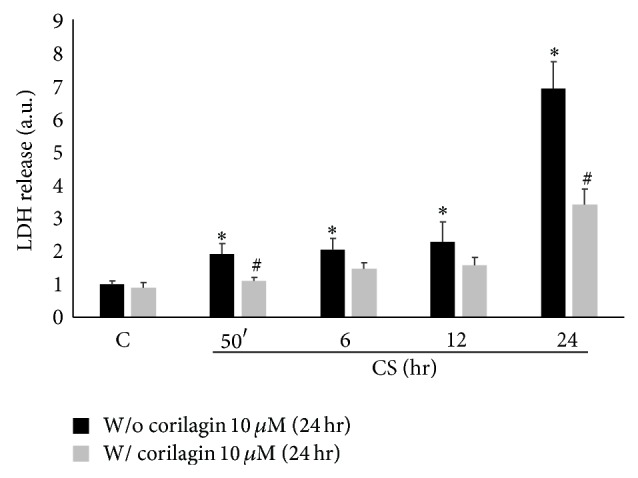
Corilagin pretreatment modulates the toxic effect of CS. The graph shows LDH release after CS exposure from cells pretreated or not with corilagin. Lower LDH levels are evident at all time points in cells pretreated. Data is representative of five independent experiments. *P* < 0.05; ∗ versus 0 hr w/o corilagin 10 *μ*M (24 hr); # versus w/o corilagin 10 *μ*M (24 hr).

The next step of our study was to investigate whether corilagin could have a protective effect against CS induced toxicity in Calu-3 cells. The dose of corilagin was chosen based on the literature and on preliminary studies where its toxicity was evaluated in the 0–100 *μ*M concentrations range (data not shown). As shown in Figure 5, corilagin pretreatment reduced about 30–50% the cytotoxicity induced by CS exposure as measured by LDH release compared to control cells (Figure 5).

### 3.6. Pretreatment with Corilagin Prevents CS Induced Loss of Cell-to-Cell Contacts

**Figure 6:**
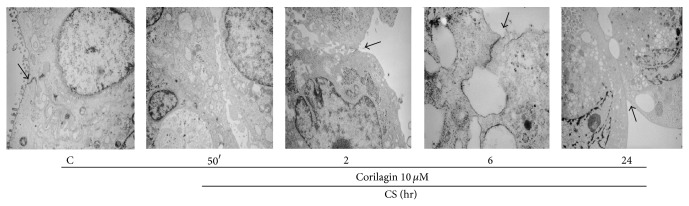
Corilagin prevents the loss of cell-to-cell contacts induced by CS. CS exposure leads to the formation of vesicles inside the cells; piled structures keeping cells together are still visible after 2, 6, and 24 hr from exposure (see black arrows). Pictures are taken at different magnifications.

As shown in Figure 6, pretreatment with 10 *μ*M of corilagin prevented CS induced loss of cell-to-cell contacts. This effect was evident at all the time points as shown by the black arrows in Figure 6.

### 3.7. Pretreatment with Corilagin Prevents CS Induced Proteins Oxidation

**Figure 7:**
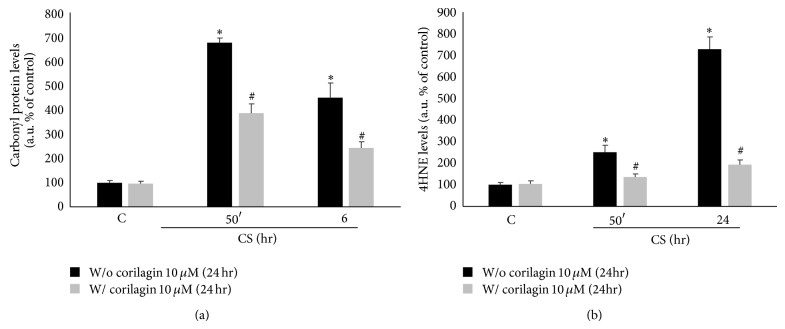
CS induced oxidative markers are modulated by corilagin. The WBs shown on the top are representative of five different experiments. Quantification of carbonyl proteins is shown in the bottom left panel (a) and quantification of 4HNE is shown in the bottom right panel (b). Data are expressed as arbitrary units (average of five independent experiments; *P* < 0.05). ∗ versus 0 hr w/o corilagin 10 *μ*M (24 hr); # versus w/o corilagin 10 *μ*M (24 hr).

Calu-3 cells pretreated with corilagin showed significantly decreased levels of carbonyl proteins induced by CS of about 50% during the analyzed time points (Figure 7(a)). The same effect was observed for the formation of 4HNE-protein adducts as shown in Figure 7(b), with a level of 4HNE almost back to the control levels in the cells pretreated with corilagin. These data confirm that corilagin operates on early CS-mediated changes (see in particular Figure 7(a)).

### 3.8. Corilagin Prevents CS Induced Connexin 40 Expression

**Figure 8:**
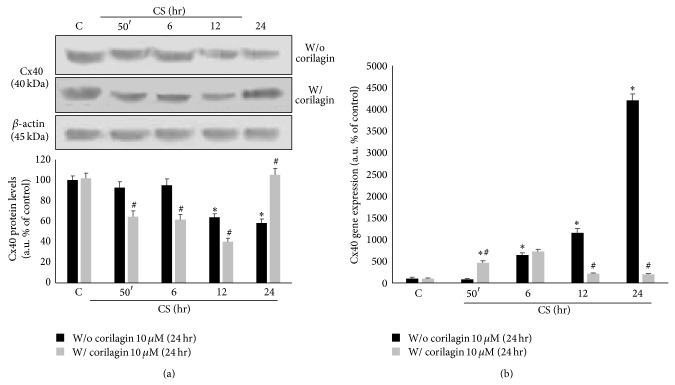
Corilagin reestablishes connexin 40 protein and gene levels after CS exposure. WBs in the top left panels are representative of five independent experiments. Bands quantification is shown in the bottom left panel (a). *β*-actin was used as loading control. Gene expression is shown in the right panel (b). Data are expressed as arbitrary units (average of five independent experiments; *P* < 0.05); ∗ versus 0 hr w/o corilagin 10 *μ*M (24 hr); # versus w/o corilagin 10 *μ*M (24 hr).

As connexin 40 (Cx40) is a protein present in cellular gap junctions, we evaluated whether its expression is affected by CS exposure in Calu-3 cells. As shown in Figure 8(a), the protein levels of Cx40 decreased upon CS exposure starting at 12 hr, while corilagin pretreatment partially prevented this decrease. In parallel, as it is depicted in Figure 8(b), Cx40 gene expression started to increase significantly in a time-dependent manner after 6 hr from CS exposure (7-fold) and it returned to the control levels at the later time points (12–24 hr) in cells pretreated with corilagin.

**Figure 9:**
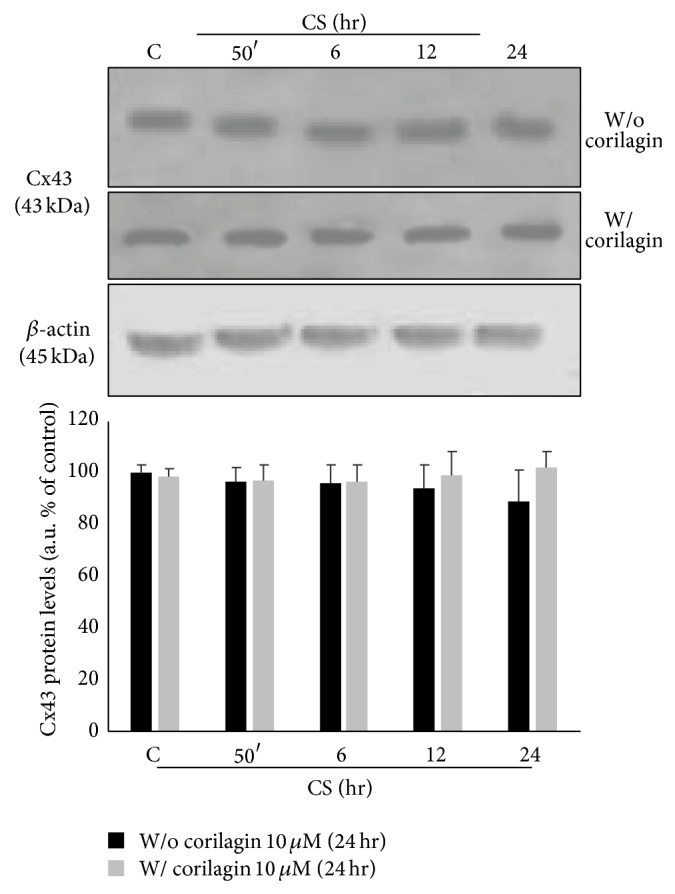
Effect of CS and corilagin in Cx43 protein levels. Top panel shows representative WBs of five independent experiments. Bands quantification is shown in the bottom panel. *β*-actin was used as loading control.

This effect was not noticed for connexin 43 (Cx43), another protein present in the gap junctions, as it is shown in Figure 9.

### 3.9. Corilagin Prevents CS Induced Cx40-4HNE Adduct Formation

**Figure 10:**
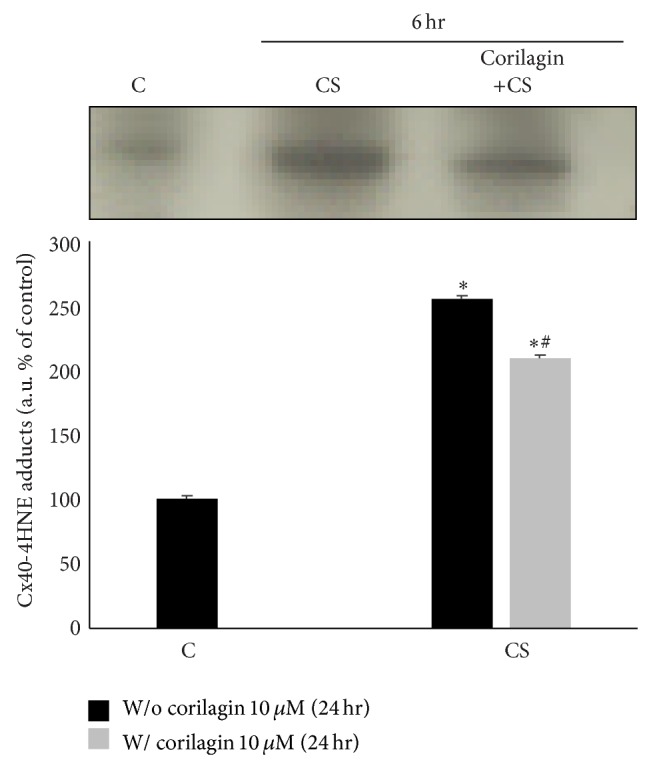
Corilagin reduces the formation of 4HNE-Cx40 adducts induced by exposure to CS. WB in the top panel is representative of five independent experiments. Bands quantification is shown in the bottom panel. Data are expressed as arbitrary units, average of five independent experiments; *P* < 0.05. ∗ versus C; # versus CS.

Since 4HNE can bind to the proteins –SH groups and Cx40 contains several cysteine amino acids (–SH group), the formation of Cx40-4HNE adducts was evaluated by immunoprecipitation upon CS exposure with/without the presence of corilagin. As shown in Figure 10, there was a clear and significant formation of 4HNE-Cx40 adducts (2.5-fold) after CS exposure and this effect was clearly prevented by corilagin pretreatment.

### 3.10. Corilagin Inhibits CS Induced NF*κ*B Activation

**Figure 11:**
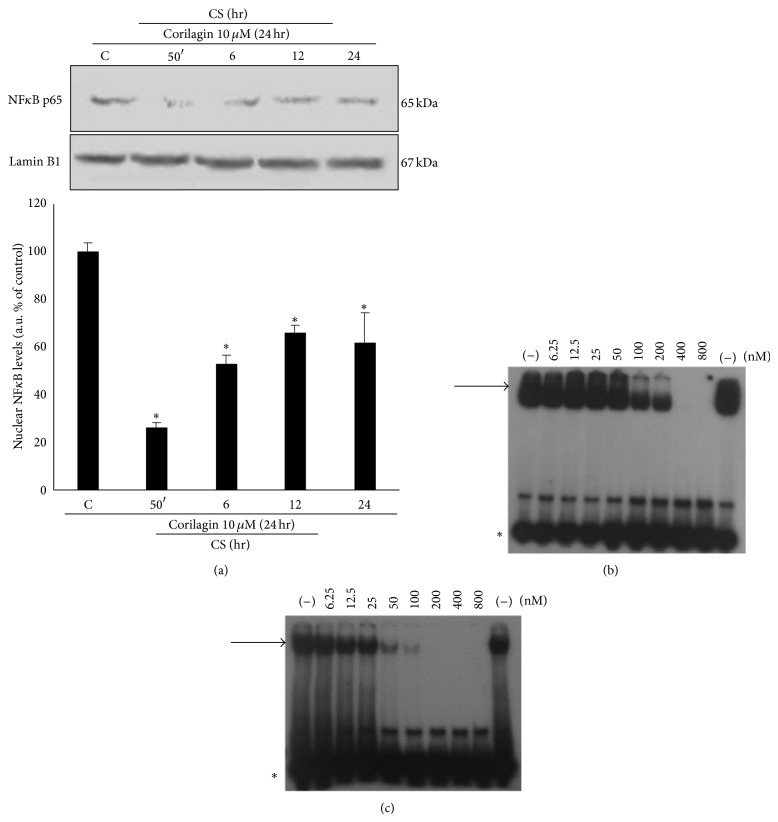
Effects of corilagin on NF*κ*B. 24 hr of pretreatment with corilagin decreases the activation of NF*κ*B induced by CS. WB shown in the top panel (a) is representative of five independent experiments. In the bottom of panel (a) is shown the quantification of nuclear NF*κ*B protein levels. Data are expressed as arbitrary units (average of five independent experiments; *P* < 0.05; ∗ versus C). Lamin B1 was used as loading control. Corilagin inhibits the interaction between NF*κ*B and oligonucleotides mimicking NF*κ*B binding sites (panel (b) NF*κ*B consensus sequence A; panel (c) NF*κ*B consensus sequence B). EMSA analysis was performed on binding reactions conducted in the presence of the indicated nM concentrations of corilagin.

The reason for analyzing the effects of corilagin on NF*κ*B was based on (a) the evidence that CS induces activation of NF*κ*B (see Figure 4) and (b) the reported finding that the promoter of the connexin 40 gene (GJA5) has NF*κ*B consensus binding sequences, suggesting a NF*κ*B dependent transcriptional regulation [33]. In addition, corilagin was reported to prevent NF*κ*B activation and regulate NF*κ*B dependent gene expression in a different experimental model system [26]; we have analyzed whether also in this cellular and experimental model corilagin could prevent CS induced NF*κ*B activation and function. Indeed, as it is shown in Figure 11, cells pretreated with corilagin showed a clear and significant inhibition of NF*κ*B activation induced by CS (4-fold at 50 min and 2-fold at 6–24 hr), as measured by p65 nuclear translocation. Furthermore, corilagin was able to inhibit, although with a slightly different efficiency, the NF*κ*B interaction to oligonucleotides mimicking the two major NF*κ*B consensus sequences (Figures 11(b) and 11(c)).

## 4. Discussion

The results presented in this study underlined the effects that CS has on lung epithelial cells, with focus on cellular junctions, and how a polyphenolic compound, corilagin, can attenuate these effects.

The respiratory tract is a complex system important for the process of respiration and it allows the direct contact between human body and external oxidative environment that can be noxious to lung tissues. Indeed, lung functionality is maintained thanks to the RTLF (respiratory tract lining fluid), which is rich in antioxidants compounds, and to the compact epithelium, composed of cells containing multiple junctions [34].

Therefore, lung epithelium, when intact, is able to protect the respiratory tract from outdoor stressors [35]. Nowadays, many studies focus on the composition of the environmental air as source of damage for human health. Pollutants such as ozone [36], ambient particles [37], and cigarette smoke (CS) [38] are among the most toxic to which living organisms are continuously exposed.

It is well documented that CS is toxic on airway cells, especially due to oxidative damage [39]. In our study we used LDH as a marker of cytotoxicity in Calu-3 cells and we observed increased levels of LDH in cells exposed to CS, confirming the noxious effect that CS has on the airways epithelium. LDH is released from cells mostly after damage to the cellular membrane and often is correlated to necrosis cellular process. In our experimental procedure, as previously published [29], the cell number did not vary in a significant manner as demonstrated by the trypan blue assay; therefore we have analyzed the cells by TEM in order to evaluate the cellular morphology changes induced by CS. Of interest was the fact that the cells exposed to CS clearly showed a loss in cell-to-cell contact. This result is in line with previous work by Upham et al. showing that CS inhibited the formation of gap junctions via the activation of extracellular receptor kinase in liver epithelial cells of WB-F344 rat [40]. In addition, a previous work has shown that oxidative stress induced by cigarette smoke extract (CSE) or H_2_O_2_ (which is also present in CS) is able to induce the opening of gap junction hemichannels: this will cause membrane depolarization and the opening of the hemichannels will facilitate the entry of toxic molecules that in turn can injure the cells [41]. In our study we were not able to detect a significant cell death but this could be just a matter of timing and CS doses. In fact, in the study of Ramachandran, the authors have used a high dose of H_2_O_2_ (1 mM) and CSE concentrated that has a quite different composition from freshly smoked CS used in our experiments. In addition, in our previous work we detected a concentration of H_2_O_2_ in CS ranging from 30 to 100 *μ*M [37], which is much lower than the one used by Ramachandra et al. [41].

Gap junctions and connexins, in particular, are important not only in cellular communication but also in cell-to-cell adhesion. Among the most representative connexins in the lung, there are connexin 40 (Cx40) and connexin 43 (Cx43) [42, 43]. We have shown that CS was able to decrease the levels of Cx40 in a time-dependent manner and this is in parallel with the induction of new Cx40 mRNA, which could be interpreted as a protective cellular response of the cells to the loss of the protein. In addition this confirms the fact that the cells were alive and able to start the new transcripts. Whether the CS-mediated effects on Cx40 and Cx43 are NF-*κ*B dependent remains to be determined.

Our work is in agreement with many other studies showing that oxidative stress, in specific CS, is able to induce the activation of NF*κ*B [44]. Its activation can be measured indirectly by analyzing the nuclear translocation of its cytoplasmic subunits among which there is p65. We have shown that the nuclear level of p65 in Calu-3 cells after CS exposure was significantly increased, demonstrating the activation of NF*κ*B by CS in airway epithelial cells. On the other hand, we have demonstrated that corilagin inhibits NF*κ*B activation and has important effects on the interactions between NF*κ*B and DNA. As far as NF*κ*B activation, it is possible that this is the consequence of CS induced oxidative stress as demonstrated by the increased levels of protein carbonyls formation and also by the increased levels of 4HNE-protein adducts. We have observed that when the cells were exposed to CS for 50 min, the levels of carbonyl proteins increased quickly, indicating that CS led to the oxidation of proteins. Similar response was observed in the case of 4HNE adducts, as CS induced lipid peroxidation and formation of *α*,*β*-unsaturated aldehydes.

Our study suggests another possible mechanism by which CS can affect gap junction besides the one elegantly proposed by Ramachandra et al.; that is, the loss of gap junction is a consequence of the formation of 4HNE-connexin adducts. In fact, since CS induced the formation of 4HNE, this aldehyde is able to bind to Cys, His, or Lys residues via Michael addition that are present in large amount in Cx40. Our data demonstrated that Cx40 was able to form adducts with 4HNE and this could be the reason of the protein loss that, once modified by 4HNE, can be degraded by the proteasome [37]. This effect seems to be related only to Cx40; in fact neither Cx43 protein levels nor its mRNA levels were affected by CS exposure. This is corroborated by a study showing that connexin expression is frequently disrupted in response to lung pathology; for instance, during the acute phase of lung injury, Cx43 expression does not decrease while Cx40 expression decreases [45].

The connexins, when present in the same cells, can form heterodimers in the gap junctions channels as demonstrated for Cx40/Cx43, Cx37/Cx43, and Cx40/Cx37 [46]; therefore it is possible that the loss of just one kind of connexin, in our case Cx40, can affect the functionality of several gap junctions.

A recent study confirms anyways the importance of Cx40 in lung structure; in fact using animal KO for Cx40 the authors have shown that the animals developed severe lung abnormalities such as fibrosis and altered alveolar remodeling [47].

In the second part of our study the possible protective effect of the polyphenol corilagin on CS induced cell damage and loss of gap junctions has been evaluated. Corilagin is a polyphenolic compound known for its strong ability of inhibiting NF*κ*B activation [26]. The results of the study confirm that corilagin inhibits CS induced NF*κ*B activation. The protective effect of corilagin against lung injury in a bleomycin model has been recently demonstrated [48] together with its ability to quench free radicals and inhibit NF*κ*B [49, 50]. In addition, pretreatment with corilagin was able to counteract CS induced protein oxidation and lipid peroxidation, as determined by carbonyl proteins formation and 4HNE proteins adducts. Furthermore, corilagin was also able to prevent the loss of Cx40, most likely thanks to its free radical quenching properties, therefore preventing the formation of Cx40-4HNE-protein adducts.

In parallel, Cx40 gene expression appeared to be less enhanced by CS when Calu-3 cells were pretreated with the polyphenol as a consequence of the effect of corilagin on preventing Cx40 protein loss. Interestingly, the promoter of connexin 40 (GJA5) has NF-*κ*B consensus binding sequences, suggesting a NF*κ*B dependent transcriptional regulation [33]. However, further work is necessary to verify whether the reduced transcription of Cx40 in the presence of corilagin is dependent on the inhibitory effects of corilagin on NF-*κ*B. In any case and whatever the molecular basis of the corilagin-mediated effects is, TEM analysis showed that treatment with corilagin prevented the loss of cell-to-cell junctions, enforcing the theory of Cx40–Cx43 heterodimers gap junctions presence in airway epithelium, since Cx43 protein expression was not affected by CS exposure. Of note is that in the present study corilagin was used at the concentration of 10 *μ*M which although used in other studies present in the literature [24, 26] could be a dose difficult to reach in the lung tissue due to its bioavailability. Therefore, we could suggest the use of corilagin by nebulization, to avoid its degradation in the GI tract and to reach higher doses in the respiratory tract.

Altogether, the results presented in our study showed that CS is able to damage airway epithelium integrity by causing cellular junctions loss through oxidative damage and NF*κ*B activation and that corilagin, thanks to its anti-inflammatory and free radical scavenging characteristics, prevents CS induced cell damage.

## Figures and Tables

**Table 1:** Cellular viability as measured by trypan blue assay.

Samples	Cell viability (%)
Control	89% ± 15
CS 50 min	78% ± 12
CS 6 hr	69% ± 21
CS 24 hr	62% ± 18
